# Geranylgeraniol and Green Tea Polyphenols Mitigate Negative Effects of a High-Fat Diet on Skeletal Muscle and the Gut Microbiome in Male C57BL/6J Mice

**DOI:** 10.3390/metabo12100913

**Published:** 2022-09-27

**Authors:** Chwan-Li Shen, Moamen M. Elmassry, Katherine Grue, Hayli E. Joiner, A. Unique Jacobo, Abdul Hamood, Eunhee Chung

**Affiliations:** 1 Department of Pathology, Texas Tech University Health Sciences Center, Lubbock, TX 79430, USA; 2 Center of Excellence for Integrative Health, Texas Tech University Health Sciences Center, Lubbock, TX 79430, USA; 3 Center of Excellence for Translational Neuroscience and Therapeutics, Texas Tech University Health Sciences Center, Lubbock, TX 79430, USA; 4 Obesity Research Institute, Texas Tech University Health Sciences Center, Lubbock, TX 79430, USA; 5 Department of Biological Sciences, Texas Tech University, Lubbock, TX 79409, USA; 6 Department of Kinesiology and Sport Management, Texas Tech University, Lubbock, TX 79409, USA; 7 Department of Immunology and Molecular Microbiology, Texas Tech University Health Sciences Center, Lubbock, TX 79430, USA; 8 Department of Kinesiology, University of Texas at San Antonio, San Antonio, TX 78249, USA

**Keywords:** geranylgeraniol, green tea polyphenol, obesity, type 2 diabetes, skeletal muscle, gut microbiota

## Abstract

Natural bioactive compounds are proposed as alternatives in mitigating obesity-associated skeletal muscle dysfunction. The objective of this study was to test the hypothesis that the combination of geranylgeraniol (GGOH) and green tea polyphenols (GTPs) can alleviate high-fat-diet (HFD)-induced muscle atrophy and alter gut microbiome composition. Male C57BL/6J mice fed an HFD were assigned to four groups (12 mice each) in a 2 (no GGOH vs. 400 mg GGOH/kg diet) × 2 (no GTPs vs. 0.5% weight/volume GTPs in water) factorial design. After 14 weeks of diet intervention, skeletal muscle and cecal samples were collected and examined. Compared to the control groups, the group that consumed a combination of GGOH and GTPs (GG + GTPs) had significantly decreased body and fat mass but increased skeletal muscle mass normalized by body weight and cross-sectional area. In soleus muscle, the GG + GTP diet increased citrate synthase activity but decreased lipid peroxidation. Gut microbiome beta-diversity analysis revealed a significant difference in the microbiome composition between diet groups. At the species level, the GG + GTP diet decreased the relative abundance of *Dorea longicatena*, *Sporobacter termitidis*, and *Clostridium methylpentosum*, and increased that of *Akkermansia muciniphila* and *Subdoligranulum variabile*. These results suggest that the addition of GGOH and GTPs to an HFD alleviates skeletal muscle atrophy, which is associated with changes in the gut microbiome composition.

## 1. Introduction

Skeletal muscle is the most important organ for whole-body glucose homeostasis [1] and is responsible for approximately 80 to 90% of insulin-stimulated whole-body glucose uptake and disposal under normal conditions [2,3]. The gut microbiome is a complex community of microbes inhabiting our bodies, and in this context, the cecum, and plays a vital role in our health. Growing evidence suggests that crosstalk between gut microbiota and skeletal muscle regulates systemic low-grade inflammation, oxidative stress, mitochondrial function, and the development of metabolic diseases [4]. Disturbances of gut microbiota have also been shown to decrease skeletal muscle mass and function [4,5]. Further studies suggested that gut microbiota modulates muscle glycogen, a key energetic substrate for prolonged exercise, thus affecting glycogen’s muscle availability and function in mice [6].

In recent years, the use of bioactive compounds has become an alternative approach to preventing and alleviating obesity-associated skeletal muscle dysfunction. Among these bioactive compounds, geranylgeraniol (GGOH) and green tea polyphenols (GTPs) are good candidates. GGOH, which is found in fruits, vegetables, and grains [7], modifies testosterone production [8] and suppresses pro-inflammatory production (monocyte chemoattractant protein-1 and interleukin-6 in adipose tissue) [9]. However, the effects of GGOH on skeletal muscle properties in the development of obesity have not been investigated. Besides GGOH, GTPs and their bioactive compounds exhibit strong antioxidant and antiobesity properties, which can regulate gut microbiota composition [10]. Furthermore, previous studies have reported the beneficial effects of green tea on obesity-induced skeletal muscle disorders [11,12,13]. For example, green tea extracts mitigate HFD-induced muscle atrophy in senescent-accelerated mouse prone-8 mice [13]. GTP treatment ameliorates metabolic abnormalities and insulin resistance by enhancing insulin signaling in the skeletal muscle of Zucker fatty rats [12]. However, the effects of GGOH and GTPs on skeletal muscle-associated parameters along with gut microbiota have not been analyzed.

In the present study, we investigated the benefits of two dietary bioactive components, GGOH and GTPs, individually and in combination, on skeletal muscle properties and the gut microbiome in HFD-fed mice. We hypothesized that the combination of GGOH and GTPs would alleviate HFD-induced muscle atrophy and alter the gut microbiome composition. Findings from this study will advance our understanding of these bioactive compounds and their effects on skeletal muscle biology in the progression of obesity in humans.

## 2. Materials and Methods

### 2.1. Animals and Treatments

Five-week-old male C57BL/6J mice were purchased from the Jackson Laboratory (Bar Harbor, ME, USA). Following a 5-day acclimation period on standard chow, all mice were fed an HFD (60% of calories from fat, catalog number: D12492, Research Diets, Inc., New Brunswick, NJ, USA) and randomly assigned to four experimental groups (*n* = 12/group, group-housed with 3 mice per cage): Control (HFD only), GG (HFD supplemented with GGOH in 400 mg/kg diet), GTPs (HFD supplemented with 0.5% weight/volume GTPs in water), and GG + GTPs group (HFD supplemented with a combination of GGOH and GTPs). This study design yielded a 2 × 2 factorial design (2 (no GGOH vs. 400 mg GGOH/kg diet) × 2 (no GTPs vs. GTPs)). The diet composition of the HFD was previously reported [14,15]. GGOH (GG-Gold™, extracted from annatto, 85% purity with the remaining 15% consisting of fatty acids, plant terpenoids, sterols, and waxes) was provided by American River Nutrition (Hadley, MA, USA). GTPs (decaffeinated with a purity of 98.5%, consisting of 65.3% epigallocatechin gallate, 19.08% epicatechin-3-gallate, 9.87% epicatechin, 4.14% epigallocatechin, and 1.54% catechin, Zhejiang Yixin Pharmaceutical Co., Ltd., Zhejiang, China) were dissolved in distilled water and prepared fresh daily [16,17]. Mice were housed under a controlled temperature of 21 ± 2 °C with a 12 h light–dark cycle. Mice were fed and watered ad libitum, and body weight, food intake, and water consumption were recorded weekly. The Texas Tech University Health Sciences Center Institutional Animal Care and Use Committee approved all handling, conditions, and experiments performed.

### 2.2. Sample Collection

After 14 weeks, mice were fasted for four hours and euthanized with isoflurane. Soleus and gastrocnemius muscles were harvested, weighed, snap-frozen in liquid nitrogen, and stored at −80 °C for later analyses. White adipose tissue (i.e., epididymal fat) was collected and weighed. The cecal samples were collected from the cecum and frozen at −80 °C for later microbiome analyses.

### 2.3. Skeletal Muscle Analyses

Gastrocnemius muscles (*n* = 3 per treatment group) were fixed with 10% phosphate-buffered formalin and stained with hematoxylin–eosin to visualize tissue architecture and measure the cross-sectional area (CSA) described previously [11]. Briefly, the CSA was measured by tracing the outer perimeter of the cell with image J (NIH) over 2000 fibers per group.

All other experiments were performed using soleus muscles, which contain a high percentage of myosin heavy-chain I isoforms (MyHCs I) and are classified as Type I fibers since Type I fibers negatively correlate with obesity and metabolic disorders [18,19]. Citrate synthase (CS) activity (CS0720; Sigma-Aldrich, St. Louis, MO, USA), cytochrome c oxidase (COX) activity (CYTOCOX1; Sigma-Aldrich, St. Louis, MO, USA)), malondialdehyde (MDA) levels (#10009055, Cayman Chemical, Ann Arbor, MI, USA), and the production of hydrogen peroxide (H_2_O_2_) were determined using an Amplex red hydrogen peroxide H_2_O_2_ assay kit (A22188: Invitrogen, Waltham, MA, USA) following the respective manufacturers’ instructions.

The expression of the skeletal muscle MyHC gene was measured by quantitative real-time polymerase chain reaction (RT-qPCR) as described previously [20]. Primers for MyHC-I, IIa, and IIx were listed previously [21]. The common reference genes including Gapdh, Hprt1, Actb, 18S, and Tbp were evaluated using M-value, and reference gene expression stability was determined using Bio-Rad CFX Manager 3.2. (Biorad, Hercules, CA, USA). Tbp was the most stable among groups, so mRNA levels were normalized to Tbp using the cycle threshold (ΔΔCT), and relative fold changes were reported compared to the Control. The primers of Tbp were forward 5′-GCCTTCCACCTTATGCTCAG-3′ reverse 5′-GTTGTTGCTGCTGCTGTTG-3′.

### 2.4. Microbiome Profiling

This was performed as previously described [22]. Briefly, stool samples were collected from mice cecum, and then microbial DNA was isolated using MoBio PowerFecal^®^ DNA Isolation Kit (MO BIO Laboratories, Inc., Carlsbad, CA, USA). The V3–V4 regions of the bacterial 16S rRNA gene were amplified using the bacterial universal primer sets 341F and 805R [23]. DNA was quantified; then, libraries were prepared using Nextera XT index kit v2 (Illumina, San Diego, CA, USA) following the manufacturer’s instructions. The libraries were then sequenced on a MiSeq sequencer (Illumina Inc., San Diego, CA, USA) using a 600-cycle v3 sequencing kit at the Center for Biotechnology and Genomics, Texas Tech University, Lubbock, TX, USA. Raw sequencing data have been deposited under BioProject accessions PRJNA551310 (for the HFD group), PRJNA637806 (for the GG group), PRJNA589473 (for the GTP group), and PRJNA843790 (for the GG + GTP group) in the National Center for Biotechnology Information (NCBI) BioProject database.

### 2.5. Statistical Analysis

#### 2.5.1. Physiological Data Analysis

The data are presented as a mean ± standard error of the mean (SEM). SigmaStat software version 14.0 (Systat Software, Inc., San Jose, CA, USA) was used for data analysis of these parameters by the two-way analysis of variance (ANOVA) test, followed by the post hoc Fisher’s Least Significant Difference (LSD) test. A significance level of *p*-value < 0.05 applies to all statistical tests. The figures were made using GraphPad Prism software version 9.0 (GraphPad, San Diego, CA, USA).

#### 2.5.2. Microbiome Data Analysis

16S rRNA gene sequencing data were analyzed using QIIME 2 [24]. Briefly, reads were filtered, denoised, and merged. Then, DADA2 plugin (within QIIME 2) was used to determine the exact amplicon sequence variants (ASVs). For taxonomy assignment, Greengenes database version 13.8 was used. For redundancy analysis, we used Calypso software, which generated the redundancy analysis (RDA) plot and measured the statistical significance of the diet effect on microbial community composition [25]. We used the nonparametric Kruskal–Wallis test followed by Dunn’s test for multiple comparisons. Results were regarded as significant when the *p*-value < 0.05.

## 3. Results

### 3.1. Final Body Weight and Fat Pad Weight

**Figure 1:**
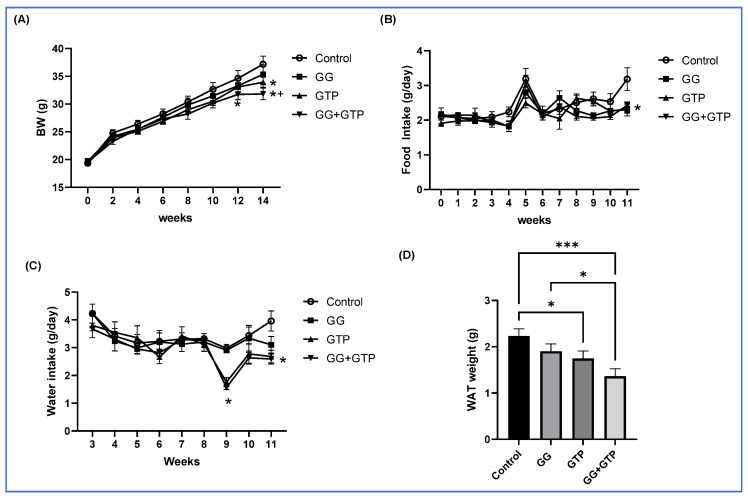
Effects of GGOH and GTPs on BW, food intake, water intake, and WAT weight in male mice fed an HFD. (**A**) Body weight, (**B**) food intake, and (**C**) water intake over time. (**D**) Final WAT weight: two-way ANOVA found no interaction effects (**A**–**D**). BW, body weight; Control, HFD only; GG, HFD supplemented with GGOH in 400 mg/kg diet; GTPs, HFD supplemented with 0.5% weight/volume GTPs in water; GG + GTPs, HFD supplemented with a combination of GGOH and GTP; WAT, white adipose tissue; *, *p* < 0.05 compared to the control; +, *p* < 0.05 compared to GG for (**A**–**C**); *, *p* < 0.05; ***, *p* < 0.001 by Fisher’s Least Significant Difference (LSD) test post hoc analysis (**D**).

We previously showed that mice fed an HFD had significantly increased body weight and fat pad weight, impaired glucose tolerance, and reduced insulin sensitivity compared to mice fed a low-fat diet [14,26]. In addition, we reported an alteration in gut microbiome composition in the HFD group compared to the low-fat-diet group [15,26]. Thus, in this study, we tested bioactive compounds in the mice fed an HFD. The initial body weight was not different across treatment groups. As shown in Figure 1A, starting at 12 weeks of intervention, mice fed a combination of GGOH and GTPs (GG + GTPs) showed a significantly lower body weight than the control group. At 14 weeks, the GG + GTPs group also showed a lower body weight compared to the group administered GGOH (GG), resulting in the order of control group = GG group > GTP group > GG + GTPs group. The overall food intake was similar among groups, except for week 11, where food intake was lower in all treatment groups compared to the control group (Figure 1B). The water intake was significantly lower with GTP administration (GTPs and GG + GTPs) in weeks 9 and 11 (Figure 1C). Two-way ANOVA factorial analysis indicated that the individual and combination of GGOH and GTP supplementation lowered white adipose tissue (WAT) weight (Figure 1D). We did not observe an interaction between GGOH and GTP supplementation on WAT weight (Figure 1D).

### 3.2. Muscle Mass and Cross-Sectional Area

**Figure 2:**
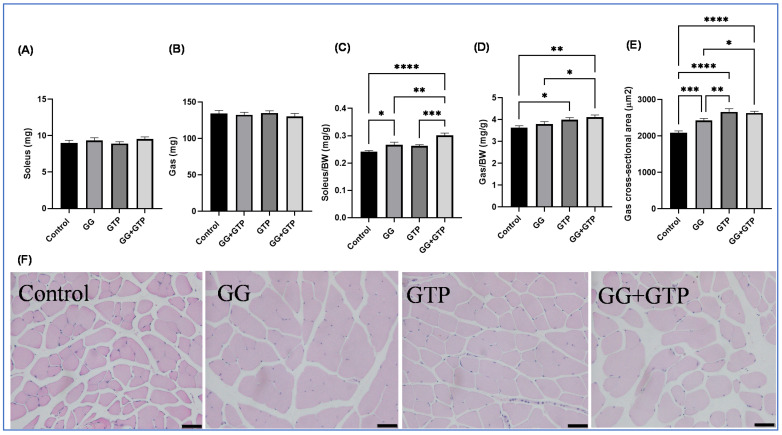
Effects of GGOH and GTPs on muscle morphology. (**A**) Soleus weight, (**B**) gastrocnemius weight, (**C**) soleus weight normalized by BW. (**D**) Gastrocnemius weight normalized by BW. (**E**) Gastrocnemius muscle CSA. (**F**) Representative images of control, GG, GTPs, and GG + GTPs gastrocnemius muscle sections stained with hematoxylin and eosin. The scale bar = 100 μm. Over 2000 fibers per group were counted. BW, body weight; Control, HFD only; GG, HFD supplemented with GGOH in 400 mg/kg diet; GTPs, HFD supplemented with 0.5% weight/volume GTPs in water; GG + GTPs, HFD supplemented with a combination of GGOH and GTPs; *, *p* < 0.05; **, *p* < 0.01; ***, *p* < 0.001, and ****, *p* < 0.0001 by Fisher’s Least Significant Difference (LSD) test post hoc analysis.

The absolute muscle weights (soleus and gastrocnemius muscles) were not changed with treatment (Figure 2A,B). However, both the GG and GG + GTPs groups had significantly increased relative soleus muscle weight (i.e., soleus muscle weight normalized to body weight) (Figure 2C). Only the GTP group, and not the GG group, had an increased relative gastrocnemius weight (i.e., gastrocnemius muscle normalized to body weight) (Figure 2D). However, the CSA of gastrocnemius muscles was significantly different, resulting in the order of GG + GTP group = GTP group > GG group > control group (Figure 2E). We did not observe the interaction between GGOH and GTP supplementation on both soleus and gastrocnemius weights of animals (Figure 2C,D), but significant interaction effects on the CSA were seen (Figure 2E). Representative images are shown in Figure 2F.

### 3.3. Mitochondrial Enzyme Activity and Oxidative Stress of Skeletal Muscle

**Figure 3:**
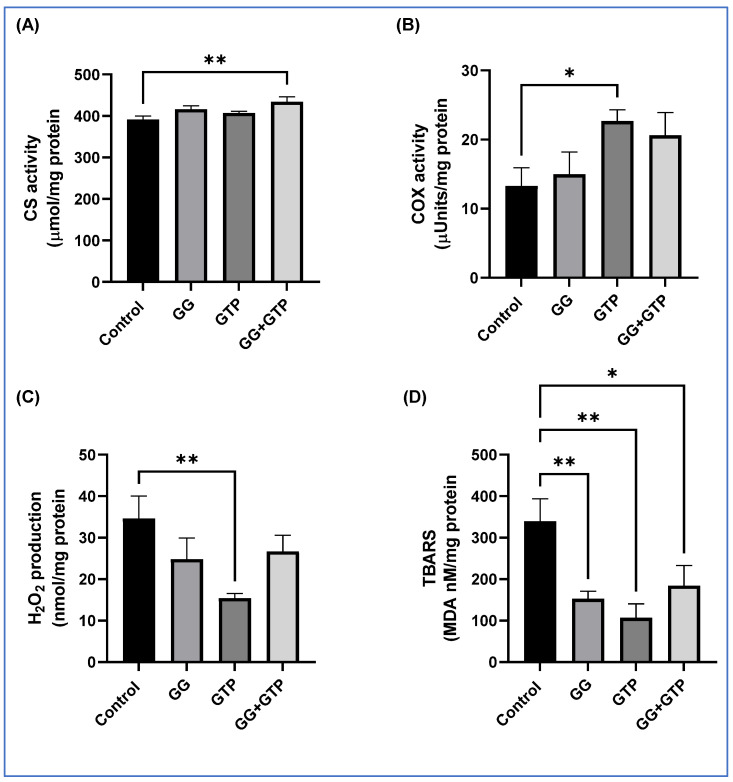
Effect of GGOH and GTPs on (**A**) CS activity, (**B**) COX activity, (**C**) H_2_O_2_ production, and (**D**) TBARS level of the soleus muscle. Values are mean (*n* = 6/group) with the standard error of the mean (SEM). CS, citrate synthase; COX, cytochrome c oxidase; Control, HFD only; GG, HFD supplemented with GGOH in 400 mg/kg diet; GTPs, HFD supplemented with 0.5% weight/volume GTPs in water; GG + GTPs, HFD supplemented with a combination of GGOH and GTPs; H_2_O_2_, hydrogen peroxidase; TBARSs, thiobarbituric-acid-reactive substances; MDA, malondialdehyde. *, *p* < 0.05; **, *p* < 0.01 by Fisher’s Least Significant Difference (LSD) test post hoc analysis.

Here, we showed the effects of GGOH and GTP supplementation on mitochondrial enzyme activity and oxidative stress of soleus muscle (Figure 3). The combination of GGOH and GTPs (GG + GTPs) significantly increased CS activity (Figure 3A). Only the GTP group, not the GG group, had significantly increased COX activity. There was no interaction between GGOH and GTP supplementation in CS and COX activity (Figure 3A,B). We measured hydrogen peroxide (H_2_O_2_) production and thiobarbituric-acid-reactive substances (TBARSs) as indirect oxidative stress markers (Figure 3C,D). There was a significant interaction between GGOH and GTP supplementation in both markers (*p* < 0.05). The GTP group had suppressed H_2_O_2_ production as well as TBARS levels. The GG group had decreased TBARS levels, and the control group had the highest H_2_O_2_ and TBARS levels among all groups (Figure 3C,D).

### 3.4. Gene Expression of Skeletal Muscle Motor Protein

**Figure 4:**
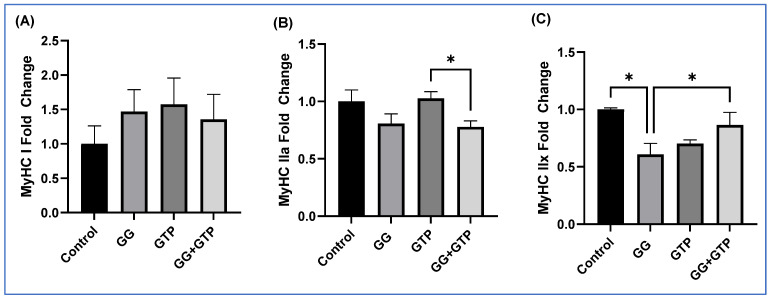
The effects of GGOH and GTP supplementation on myosin heavy-chain isoform mRNA gene expression in soleus muscle. (**A**) MyHC-I, (**B**) MyHC IIa, (**C**) MyHC IIx. *n* = 4 control; *n* = 5 GG, GTPs, and GG + GTPs, respectively. Control, HFD only; GG, HFD supplemented with GGOH in 400 mg/kg diet; GTPs, HFD supplemented with 0.5% weight/volume GTPs in water; GG + GTPs, HFD supplemented with a combination of GGOH and GTPs; MyHC, myosin heavy chain; *, *p* < 0.05 by Fisher’s Least Significant Difference (LSD) test post hoc analysis.

The impact of GGOH and GTP supplementation on MyHC isoforms of the soleus muscles was determined (Figure 4A–C). There was no effect on the expression of MyHC-I (Figure 4A). There was a significant difference between the GTP group and the GG + GTP group, but no interaction was found in mRNA expression of MyHC-IIa (Figure 4B). The GG group had significantly decreased mRNA expression of MyHC-IIx compared to the control group, while the GG + GTP group had significantly increased levels compared to the GG group with significant interaction effects (Figure 4C).

### 3.5. Gut Microbiome Diversity and Composition

**Figure 5:**
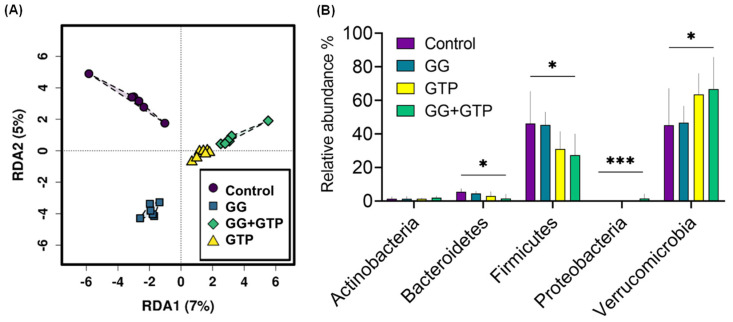
Microbiome composition overview of different diet groups. (**A**) Beta-diversity multivariate analysis using redundancy analysis (RDA). (**B**) Relative abundance differences in major phyla among diet groups. Control, HFD only; GG, HFD supplemented with GGOH in 400 mg/kg diet; GTPs, HFD supplemented with 0.5% weight/volume GTPs in water; GG + GTPs, HFD supplemented with a combination of GGOH and GTPs; * *p* < 0.05 and *** *p* < 0.001. Kruskal–Wallis test was performed followed by Dunn’s test.

Microbiome alpha diversity (i.e., microbiome richness and evenness) did not differ with different supplementations. Beta diversity (i.e., a measure of the similarity or dissimilarity of microbiome communities) was examined using redundancy analysis, which revealed a significant difference in the gut microbiome composition based on diet supplementation (Figure 5A). The most abundant phyla were Verrucomicrobia and Firmicutes among all treatment groups, while Bacteroidetes, Actinobacteria, and Proteobacteria were detected at a much lower abundance (Figure 5B). While the relative abundance of Firmicutes decreased in the GG + GTP group, the relative abundance of Verrucomicrobia increased in the GG + GTP group. Such an effect on Bacteroidetes and Proteobacteria was observed with the same pattern in the GG + GTP group (Figure 5B).

**Figure 6:**
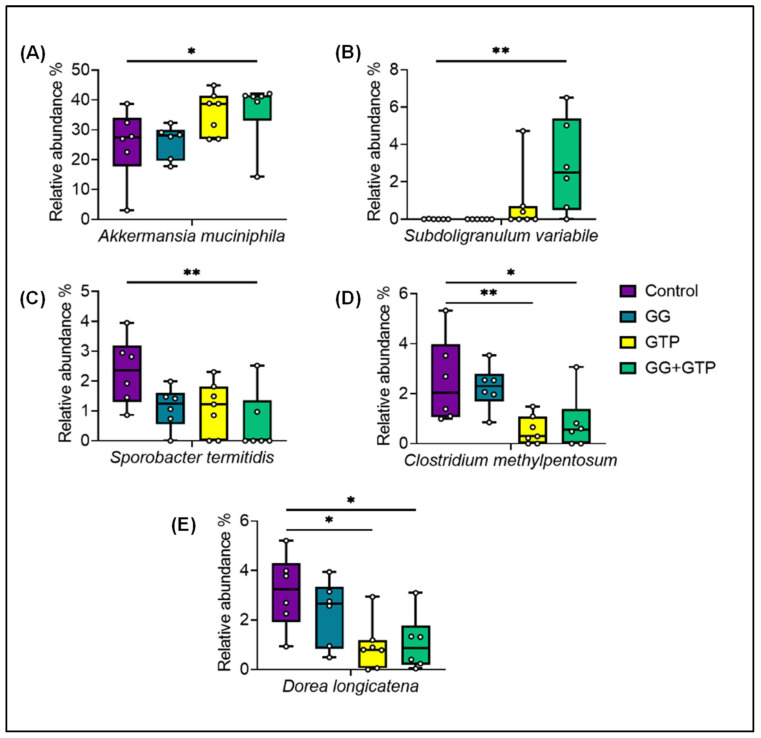
Amplicon sequence variants (ASVs) with a statistically significant differential abundance of (**A**) *Akkermansia muciniphila*, (**B**) *Subdoligranulum variabile*, (**C**) *Sporobacter termitidi*, (**D**) *Clostridium methylpentosum,* and (**E**) *Dorea longicatena* among diet groups. Control, HFD only; GG, HFD supplemented with GGOH in 400 mg/kg diet; GTPs, HFD supplemented with 0.5% weight/volume GTPs in water; GG + GTPs, HFD supplemented with a combination of GGOH and GTPs. * *p* < 0.05 and ** *p* < 0.01. Kruskal–Wallis test was performed followed by Dunn’s test.

From the microbiome analysis of mice ceca, we observed changes in the abundance of several species (Figure 6). While the individual groups of GG or GTPs affected the relative abundance of a few species, a stronger and statistically significant effect was observed with the combined treatment (the GG + GTP group) and in some cases with the GTP group only. The GTP group decreased the abundance of *Clostridium methylpentosum* and *Dorea longicatena*. Compared to the control group, the effect of GG + GTPs increased the relative abundance of *Akkermansia muciniphila* (*A. muciniphila*) and *Subdoligranulum variabile* (*S. variabile*) and decreased the abundance of *Sporobacter termitidis* (Figure 6).

## 4. Discussion

Accumulating evidence suggests that skeletal muscle atrophy associated with metabolic diseases is highly linked to oxidative stress, mitochondrial dysfunction, and dysbiosis (i.e., imbalanced gut microbiota) [4,27,28,29]. Supporting our hypothesis, mice fed an HFD supplemented with GGOH and GTPs for 14 weeks had independent benefits, while the combination of GGOH and GTPs (GG + GTP group) rather than individual supplementation had much greater effects on the muscle mass, fat mass, oxidative stress markers, and gut microbiome composition. This is the first study showing that supplementation of GGOH and GTPs for 14 weeks in mice fed an HFD could alter skeletal muscle properties and gut microbiome composition.

The absolute weights were not altered, which agrees with previous studies [18,30]. Absolute skeletal muscle mass can be attributed to extracellular matrix content and intramuscular adipose tissue, which significantly increases with obesity [31]. We believe the cross-sectional data in addition to relative muscle mass demonstrate the positive effects of GG and GTPs on skeletal muscle mass. The observations that GGOH increased skeletal muscle weight are corroborated by previous work. For example, a low dose of GGOH increases the CSA of muscle by suppressing atrogin-1 expression in denervation-induced muscle atrophy [32]. GGOH administration has also been shown to upregulate testosterone synthesis in testis-derived cells [33], which can promote muscle hypertrophy by suppressing the expression of atrogin-1 and MuRF-1 [34,35]. The increased muscle mass by GTP supplementation shown in this study is supported by previous studies. For example, GTP extract protects against endoplasmic reticulum stress, oxidative stress, and protein degradation in HFD-induced muscle atrophy [36]. The administration of epigallocatechin-3-gallate (EGCG), the most abundant catechin in GTPs, to mice significantly increased muscle fiber size after muscle damage by induction of myogenic markers, including myogenin and muscle creatine kinase [37]. GTPs mitigate HFD-induced muscle atrophy in senescence-accelerated mice [13].

Interestingly, the GG + GTP group had much larger increases in muscle mass than the GG group despite previous studies showing that GGOH can upregulate testosterone synthesis [33]. The dose of GGOH used in this study may not be sufficient to activate hypertrophic pathways or inhibit the atrophic pathways [32], but may regulate inflammation [38]. It is accepted that crosstalk between adipose tissue and skeletal muscle determines muscle quality [39]. Inflammatory cytokines and hormones released by adipose tissue in the states of both obesity and metabolic diseases negatively affect muscle mass [40,41]. In contrast, myokines induced by skeletal muscle through exercise training decrease leptin and inflammatory cytokines in circulation, which contributes to decreased adipose tissue size [42]. We previously reported that GTPs increased the fat-free mass and decreased fat mass in HFD-induced obese rats [16,43]. Based on previous studies, we can speculate that the antioxidant and anti-inflammatory effects of GTPs [44] with GGOH administration may result in the highest muscle mass (i.e., GG + GTPs group), which may then contribute to the WAT mass.

Obesity is also associated with reduced skeletal muscle oxidative capacity [45,46] and mitochondrial dysfunction is a quite common pathology in metabolic disorders [28]. CS activity, a biomarker for mitochondrial density in skeletal muscle [47], was increased, and the level of TABARS, a lipid peroxidation maker, was decreased when used in a combination (i.e., GG + GTP group) in agreement with a previous study on dystrophic (i.e., mdx) mice [48]. The increased COX activity and decreased H_2_O_2_ production by GTP supplementation seen in our study also agree with a previous study in human cultured neurons and astrocytes treated with EGCG [49] and obese mice treated with GTPs [11]. Therefore, our results suggest that GTPs and GGOH can protect from obesity-associated oxidative stress, but mitochondrial functional assessment warrants future study.

Skeletal muscles are heterogeneous and composed of different fiber types based on myosin heavy-chain (MyHC) expression (i.e., Type I, IIa, and IIx) [50]. Type I fibers are more insulin-sensitive and have a higher oxidative capacity than Type II fibers [51], and thus, are more susceptible to metabolic diseases [19,52,53]. A decreased number of Type I fibers was shown in subjects with insulin resistance [52] and obesity [19]. Although our results indicate that fiber-type alterations can occur with the use of bioactive compounds in an obesogenic environment, the functional changes due to fiber-type alteration warrant further study. In addition, most skeletal muscles are hybrid, expressing mixed fibers (i.e., I/IIa, IIa/IIx, etc.). Therefore, a study on single-fiber proteomics instead of whole muscle lysates would likely provide muscle pathophysiology [54].

The species *A. muciniphila* is well-known for its beneficial properties against metabolic syndrome and has been validated in studies using rodents and humans [55]. *A. muciniphila* is more abundant in physically active women than in either sedentary people [56] or healthy subjects [57]. Improved glucose homeostasis by metformin treatment, the most used antidiabetic drug, in diet-induced obese mice was highly correlated with increased *A. muciniphila* [58,59]. Supplementation with *A. muciniphila* improved metabolic parameters in overweight/obese insulin-resistant humans [60]. Concord grape polyphenols [61] and epigallocatechin gallate from green tea increased *A. muciniphila* with improved metabolic outcomes. We did not observe any changes in the gut microbiome in the GG-treated group. Like muscle data, the dose we used in this study may not be sufficient to alter gut microbiome composition. Previously, we showed a 800 mg/kg diet of GGOH (vs. 400 mg/kg diet in this study) increased the relative abundance of *Butyricicoccus pullicaecorum* and decreased the abundance of *Dorea longicatena* compared to the control group. Thus, it is imperative to investigate the dose–response test. *S. variabile*, another butylate-producing species, was increased with GTPs in high-fat-diet-induced insulin-resistant mice [22]. An increased abundance of *S. variabile* was highly correlated with HDL cholesterol levels and negatively correlated with fat mass and insulin resistance in humans [62]. Previous studies suggested that butyrate-producing bacterial species, such as *A. muciniphila* and *S. variabile,* regulate skeletal muscle metabolism and function [63]; thus, the validation of causality warrants future study.

Interestingly, the combination of GGOH and GTPs (GG + GTP group) produced much greater effects on skeletal muscle, WAT, and gut microbiome composition than individual supplementation (GG group or GTP group). Sishi et al. [29] reported that diet-induced obesity increased oxidative stress and low-grade inflammation, which activate atrophic signaling pathways and apoptosis in skeletal muscle. GGOH may act as an antioxidant, as shown in lower TBARS levels in our study, or an anti-inflammatory, as previously shown in lipopolysaccharide-induced inflammatory responses [38]. Thus, a low dose of GGOH could act synergistically in the presence of GTPs. Supporting our speculation, previous studies show that GTPs can act as pro-oxidants with the coadministration of tocotrienol, another antioxidant [11,64]. Thus, further investigation is needed to establish a dose relationship, particularly when supplementations are coadministered.

There are some limitations to consider: (1) The CSA was measured on gastrocnemius, while the other experiments were performed on the soleus muscle. Obesity and its associated diseases result in decreased oxidative Type I fibers, which have a higher glucose handling and oxidative capacity, with a proportional increase in fast-twitch fibers, such as IIx fibers, which are glycolytic [51]. Thus, we investigated the biochemical properties using soleus muscles. However, the mass of soleus muscle was only 7–12 mg in our studied mice, so it was not feasible to perform all experiments with soleus muscle. (2) We noted that there was a peak in food intake around the fifth week and a sharp decline in the amount of drinking water consumed around the ninth week. Such changes were consistent among all animals studied, regardless of group, with the same trend during the same period. Since this study had a 14-week feeding period, changes in food intake and water consumption in the middle of the feeding experiment should not have affected the research questions we wished to address. (3) Finally, it would be informative if we could make correlations between skeletal muscle size with the changes in gut microbiome species. However, the sample size was not sufficient to determine a correlation.

In conclusion, we demonstrated that geranylgeraniol and green tea polyphenols mitigate the negative effects of a high-fat diet on adipose tissue, skeletal muscle atrophy, and the gut microbiome.

## Data Availability

Not applicable.

## Abbreviations

CS, citrate synthase activity; COX, cytochrome c oxidase activity; CSA, cross-sectional area; H_2_O_2_, hydrogen peroxide; HFD, high-fat diet; GG, HFD supplemented with GGOH in 400 mg/kg diet; GGOH, geranylgeraniol; GTPs, green tea polyphenols as well as the experimental group of HFD supplemented with 0.5% vol/wt GT in water; GG + GTPs, HFD supplemented with a combination of GGOH and GTPs; MDA, malondialdehyde; TBARSs, thiobarbituric-acid-reactive substances.
